# Treatment Delay and Clinical Outcomes in Patients with ST-Segment Elevation Myocardial Infarction during the COVID-19 Pandemic

**DOI:** 10.3390/jcm10173920

**Published:** 2021-08-30

**Authors:** Tomasz Tokarek, Artur Dziewierz, Krzysztof Piotr Malinowski, Tomasz Rakowski, Stanisław Bartuś, Dariusz Dudek, Zbigniew Siudak

**Affiliations:** 1 Department of Cardiology and Cardiovascular Interventions, University Hospital, 2 Jakubowskiego St., 30-688 Krakow, Poland; adziewierz@gmail.com (A.D.); mcrakows@cyf-kr.edu.pl (T.R.); mbbartus@cyf-kr.edu.pl (S.B.); 2 Center for Intensive Care and Perioperative Medicine, Jagiellonian University Medical College, 31-008 Krakow, Poland; 3 2nd Department of Cardiology, Jagiellonian University Medical College, 31-008 Krakow, Poland; krzysztof.piotr.malinowski@gmail.com (K.P.M.); mcdudek@cyfronet.pl (D.D.); 4 Faculty of Medicine and Health Science, Jan Kochanowski University, 25-369 Kielce, Poland; zbigniew.siudak@gmail.com

**Keywords:** COVID-19, registry, SARS-CoV-2, ST-segment elevation myocardial infarction (STEMI), mortality

## Abstract

Pandemic-specific protocols require additional time to prepare medical staff and catheterization laboratories. Thus, we sought to investigate treatment delay and clinical outcomes in COVID-19 positive and negative patients with ST-segment elevation myocardial infarction (STEMI) treated with percutaneous coronary intervention (PCI) during on- and off-hours. All consecutive patients with STEMI treated with PCI between 1 March and 31 December 2020 were enrolled in the analysis. A propensity score match was used to compare COVID-19 positive and negative patients for on- and off-hours. The study group was comprised of 877 paired patients treated during regular hours (every day 7:00 a.m. to 16:59 p.m.) and 418 matched pairs with PCI performed during off-hours (every day 17:00 p.m. to 06:59 a.m.) (ORPKI Polish National Registry). No difference in periprocedural mortality was observed between the two groups (on-hours: COVID-19 negative vs. COVID-19 positive: 17 (1.9%) vs. 11 (1.3%); *p* = 0.3; off-hours: COVID-19 negative vs. COVID-19 positive: 4 (1.0%) vs. 7 (1.7%); *p* = 0.5). Additionally, a similar rate of periprocedural complications was reported. Patients diagnosed with COVID-19 were exposed to longer time from first medical contact to angiography (on-hours: 133.8 (±137.1) vs. 117.1 (±135.8) (min); *p* = 0.001) (off-hours: 148.1 (±201.6) vs. 112.2 (±138.7) (min); *p* = 0.003). However, there was no influence of COVID-19 diagnosis on mortality and the prevalence of other periprocedural complications irrespective of time of intervention.

## 1. Introduction

The COVID-19 pandemic negatively affected access to healthcare system and timeline of treatment [1,2,3,4]. The distribution of medical attention and resources with inevitable delays in treatment related to mandatory infection control might have a detrimental impact on outcomes. Regardless of COVID-19 status, ST-segment elevation myocardial infarction (STEMI) requires rapid treatment, and percutaneous coronary intervention (PCI) is still the gold standard of care [5,6,7]. However, the fear of contamination might prevent access to the emergency system. Furthermore, pandemic-specific protocols require additional time to prepare medical staff and the catheterization laboratory before the procedure [1,2,3,4]. Thus, patients with STEMI might be exposed to longer delays for angiography with revascularization and increased risk of death [4,8,9,10,11,12]. Furthermore, some data suggest additional treatment delay for PCIs performed during off-hours [13,14]. Importantly, patients with STEMI are associated with more extensive use of potent antithrombotic and antiplatelet treatment. These agents might be linked with a higher rate of bleeding and vascular complications [5,6]. However, current reports emphasize hypercoagulability and high thrombus burden with potentially detrimental outcomes in patients with STEMI and COVID-19 diagnosis [8,9,10,11,12]. Irrespective of the exponential growth of evidence, there are still limited data on the clinical outcomes in STEMI with diagnosed COVID-19. Furthermore, there is a lack of analysis depending on the time of PCI during the COVID-19 pandemic. Thus, we sought to investigate treatment delay and clinical outcomes in COVID-19 positive and negative patients with STEMI treated during regular and nonregular hours of work.

## 2. Materials and Methods

**Figure 1:**
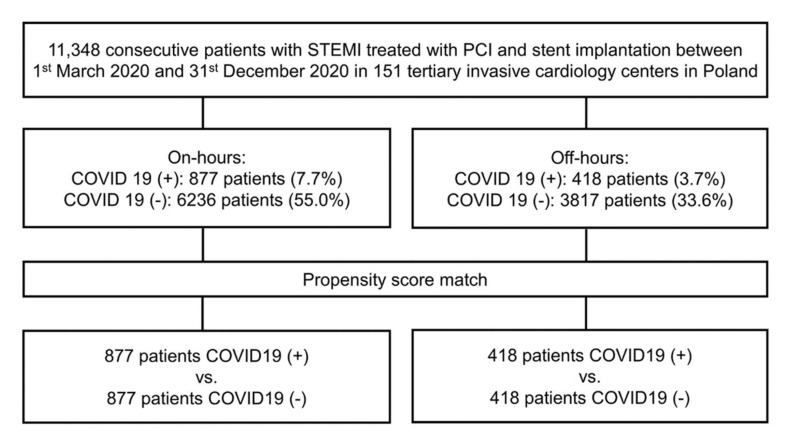
Flowchart of included patients. PCI—percutaneous coronary intervention; STEMI—ST-segment elevation myocardial.

A comprehensive delineation of the ORPKI national registry was demonstrated in previous analyses [15,16,17,18]. In brief, this registry provides evidence of all PCI procedures performed in Poland since 2004. This database is certified by the Polish Association of Cardiovascular Interventions of the Polish Cardiac Society and operated by the Jagiellonian University Medical College in Krakow [19]. Anonymized data are stored with the use of a dedicated case report form. The system structure and geographical distribution, along with the total volume of PCI, of invasive cardiology centers in Poland have been presented previously [20]. The first patient with a positive COVID-19 diagnosis was confirmed in Poland in March 2020. Thus, clinical and procedural data for this study were gathered prospectively between 1 March 2020 and 31 December 2020 from a system of 151 invasive cardiology facilities on Polish territory. A total of 11,348 consecutive patients with STEMI treated with PCI and stent implantation during on- and off-hours were enrolled in the analysis. None of the patients received fibrinolytic therapy. Patients were distributed into two groups as follows: on-hours (every day 07:00 a.m.–04:59 p.m.); off-hours (every day 05:00 p.m.–06:59 p.m.). Working frames were set in accordance with timetables and quotidian clinical practice in participating centers during the pandemic. Before the procedure, a real-time reverse transcription–polymerase chain reaction test was performed in patients to confirm COVID-19 status. The PCI procedures were conducted in 10,053 (88.6%) COVID-19 positive and 1295 (11.4%) COVID-19 negative patients, respectively. A flowchart of the included patients and their allocation into subgroups is presented in Figure 1.

To overcome potential preselection bias related to the non-randomized design, a propensity score match (PSM) was used to compare COVID-19 positive and negative patients between the two timeframes. All procedures were conducted by invasive cardiologists with diverse experience and excellence level in PCI with radial approach (RA). The total radial volume was calculated independently for each physician with the use of personal identification numbers in the ORPKI database. This was defined as the overall number of PCIs with RA use between 2014 and 2020. Furthermore, this parameter was also quantified as percentage of all PCIs conducted via RA. Both access site and target lesion selection and treatment strategy were left to the operator’s discretion. A vascular access site was defined as the site of successful entry. All procedures conducted with unknown or altered access sites were ruled out from the analysis. Additionally, detailed information on complexity and lesion morphology was not collected. All procedures were conducted following local standards of PCI and European Society of Cardiology (ESC) guidelines wherever applicable. All periprocedural complications were collected prospectively, including stroke, access-site-related bleeding, allergic reaction, no-reflow phenomenon, dissection of the coronary artery, cardiac arrest, and coronary artery perforation. All adverse events were diagnosed by local medical doctor in compliance with contemporary definitions in ESC guidelines [6]. There was no observation beyond discharge from the hospital. Periprocedural mortality was defined as death by any cause reported between PCI and transfer from catheterization laboratory to either the cardiology department or intensive treatment unit. Normalized bleeding delineation from the consent document of Bleeding Academic Research Consortium were applied in all participating facilities as any evident sign of blood lose (e.g., more bleeding than might be anticipated for a clinical context, including extravasation found by imaging alone) that is not in accordance with the criteria for type 3, 4, or 5 but conform to at least one of the following pattern: (1) demanding nonsurgical medical treatment by a healthcare specialist, (2) leading to hospital admission or greater level of care, or (3) prompting evaluation [21]. Cerebrovascular complications were diagnosed in accordance with the specialist opinion of local medical doctor based on clinical status. No further neurological evaluation was collected. Cardiac arrest was determined as the abrupt loss of organized electrical activity of cardiomyocytes with the absence of contraction of the ventricles and inability to provide an effective cardiac output. The study was asserted by the institutional ethical board. All enrolled patients supplied signed informed consent for the PCI procedure. The study was conducted in consensus with ethical principles for clinical research by virtue of on the Declaration of Helsinki with later amendments. There was no financial support for this registry.

### Statistical Methods

A propensity score was computed to mimic randomization and avoid the plausible effect of preselection bias. A multivariate logistic regression model was calculated separately for on- and off-hours with COVID-19 status (positive versus negative) set as the dependent variable. All baseline characteristics (gender, age, weight, diabetes mellitus, previous stroke, previous myocardial infarction, previous PCI, previous coronary artery bypass grafting (CABG), smoking status, arterial hypertension, chronic kidney disease, psoriasis, Killip-Kimball class on admission, cardiac arrest and hypothermia at baseline, periprocedural treatment: access site, aspiration thrombectomy, rotational atherectomy, acetylsalicylic acid, P2Y_12_ inhibitors, unfractionated heparin, low-molecular-weight heparin) and baseline clinical data (Thrombolysis in Myocardial Infarction (TIMI) scale before PCI, operator experience using radial approach in PCI procedures, direct transport, time from first medical contact to angiography and site volume ≥400 PCIs) were set as covariates. Nearest neighbor method was used to attain adequate balance in standardized differences for all confounding factors gauged as below 10%. All patients were paired in 1:1 proportion. Unmatched patients were not involved in the analysis. Typical descriptive statistics were executed in the analysis. Quantitative variables are shown as mean and standard deviation. Qualitative values are shown as counts and percentages. The Mann–Whitney U test (for non-normally distributed data) or the Student’s *t*-test (for normally distributed data) for continuous variables and the Fisher’s exact test or the Pearson’s chi-squared test for qualitative (nominal and dichotomous) data were exploited to juxtapose groups before matching. The normality of the data was evaluated with the Shapiro–Wilk test for sample sizes below 2000, and the Kolmogorov–Smirnov test with Lillieforce emendation was calculated for groups over 2000. Matched pairs of patients were compared with the Wilcoxon signed-rank test (for non-normally distributed data difference) or the paired *t*-test (for normally distributed data difference) for continuous variables and the McNemar-Bowker’s test for categorical (nominal) variables. Two-sided *p*-values < 0.05 were perceived statistically significant. All statistical analyses were computed with JMP^®^, Version 15.2.0 (SAS Institute Inc., Cary, NC, USA) and R version 4.0.4 (R Core Team, Vienna, Austria) with package MatchIt 3.0.2.

## 3. Results

**Figure 2:**
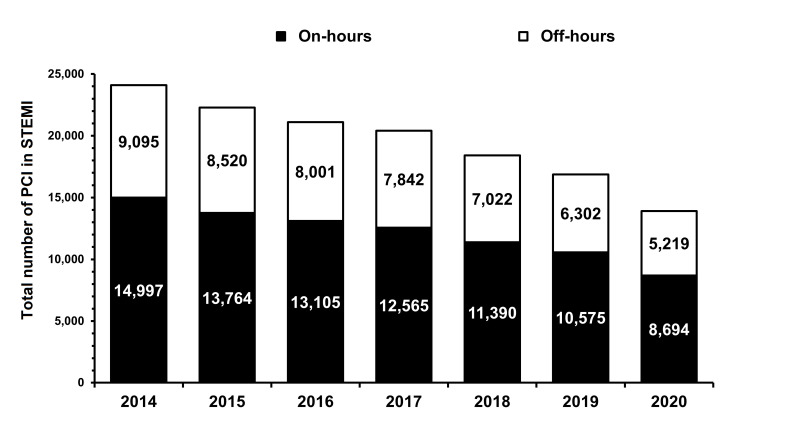
Total number of hospitalizations for PCI in STEMI for both on- and off-hours (*p* = 0.001). PCI—percutaneous coronary intervention; STEMI—ST-segment elevation myocardial.

A significant decline in the total number of hospitalizations for PCI in STEMI was observed in 2020 as compared to the previous year for both timeframes (Figure 2).

Complete baseline clinical and demographic data are presented in Table 1.

The study group was comprised of 877 paired patients treated during regular hours and 418 matched pairs with PCI performed off-hours. Both COVID-19 positive and negative patients were well matched and there were no disparities in baseline characteristics. However, patients diagnosed with COVID-19 were admitted with cardiac arrest more frequently as compared to COVID-19 negative patients during regular working hours (on-hours vs. off-hours, respectively: COVID-19 positive vs. COVID-19 negative: 180 (20.5%) vs. 64 (7.3%); *p* = 0.001; COVID-19 positive vs. COVID-19 negative: 21 (5.0%) vs. 20 (4.8%); *p* = 1.0). Similar extent of coronary artery disease in angiography as well as TIMI flow grades before and after PCI were reported during off-hours. Conversely, single-vessel disease and multivessel disease with left main coronary artery involvement were more common in the COVID-19 positive group during regular working hours. Furthermore, there was a higher percentage of patients with final TIMI flow grade 0 or 1 in this clinical setting (Table 2).

During regular working hours patients with diagnosed COVID-19 were more frequently transferred to high-volume centers. A similar trend was observed in off-hours; albeit without statistical significance (Table 3). Invasive cardiologists with comparable dexterity and experience in RA performed PCI in COVID-19 positive group during on-hours (Table 3).

There was a higher rate of ticagrelor and prasugrel use during PCI in COVID-19 positive patients but only during on-hours. Data of both antiplatelet and antithrombotic treatment during the procedure is presented in Table 3. There were no differences in radiation doses and the total contrast media load between COVID-19 positive and negative patients during both on- and off-hours (Table 2). Furthermore, a similar prevalence of any periprocedural complications was detected despite time of intervention. Additionally, periprocedural mortality did not differ between the two groups (on-hours vs. off-hours, respectively: COVID-19 negative vs. COVID-19 positive: 17 (1.9%) vs. 11 (1.3%); *p* = 0.3; COVID-19 negative vs. COVID-19 positive: 4 (1.0%) vs. 7 (1.7%); *p* = 0.5). All periprocedural outcomes are presented in Table 4. Total number of each periprocedural complication might not be equal to the “any periprocedural” complication endpoint, as one patient might experience more than one complication.

However, COVID-19 positive patients were exposed to a longer time from first medical contact to angiography during both on-hours and off-hours (Table 5).

Additionally, COVID-19 positive patients were less likely to achieve angiography <90 and <120 min during both on- and off-hours (Table 5). Time from chest pain onset to first medical contact remained similar in COVID-19 positive and negative patients during both working frame hours (Table 5).

## 4. Discussion

The main finding of our study suggests that patients with a diagnosis of COVID-19 might be exposed to a longer time to achieve angiography in STEMI during both on- and off-hours. However, COVID-19 positive patients were not associated with an increased risk of periprocedural mortality or complication irrespective of the time of intervention. To the best of our knowledge, we present the first national registry of STEMI treatment depending on the time of PCI during the COVID-19 pandemic. Several studies have confirmed the reduced number of STEMI patients regardless of geographical location [1,2,3,10,11,12,22,23,24,25]. Some authors have pointed out lower air pollution or less work-related stress during the lockdown [2,10,12]. However, decline in the number of patients with STEMI was detected immediately after COVID-19 outbreak with analogical outcomes in rural areas. Thus, the effect of air quality seems to be negligible [10,25]. Similarly, the previous study suggested a two-fold increase in stress index level in the population during the lockdown. Therefore, this explanation is also unlikely [10,25,26]. Elucidation of this data might involve many factors. However, observed results seem to be related to dismay of COVID-19 exposure or limited attainability to overwhelmed health service. Furthermore, atypical presentation of STEMI or overlapped respiratory symptoms might confuse patients and dissuade or delay seeking medical care [10,12,13]. Another plausible explanation for this phenomenon might be related to an increased rate of potentially fatal prehospital sudden cardiac arrest as compared to the previous year [10,12,25,27]. Our findings are consistent with this disturbing data. However, COVID-19 positive patients experienced this complication only during on-hours. Some evidence suggested a diurnal alteration in myocardial perfusion as a possible mechanism for a higher prevalence of STEMI during morning hours [1,6,13,14]. Regardless of the reason for the delay, longer time-to-presentation and revascularization is linked with a higher risk of cardiac arrest and death [6]. Each 30 min delay to PCI was postulated to increase relative risk for in-hospital mortality by up to 20–30% [12,13,28]. Both patient-related and systemic factors might be responsible for this phenomenon. Pandemic-specific protocols for preparing personal protective equipment and obligatory COVID-19 testing consume additional time and extend the time for reperfusion [1,2,3,4,10,12]. Most previous studies have suggested longer total ischemia time and time from first medical contact do angiography in patients admitted during COVID-19 pandemic [1,25,29]. Furthermore, some investigators have also demonstrated an escalated risk of death and adverse events [8,25,29]. However, retrospective nature without balanced rate of risk factors and low sample size might limit the generalizability of these studies. Finally, a previous meta-analysis confirmed no difference in in-hospital mortality between patients with STEMI before and during the COVID-19 pandemic [12]. Furthermore, prolonged door-to-balloon time and similar time from the symptoms onset to first medical contact were observed in patients with STEMI hospitalized during COVID-19 pandemic [12]. However, there was a potential bias related to included centers with the extensive encumbrance of COVID-19 patients with the most delayed time to treatment. Local factors might have a substantial effect on the global results of this analysis. Furthermore, this meta-analysis included only studies published before August 2020. Evidence and proficiency in the initial stage of the outbreak were limited. Global adaptation of emergency processes is evolving, and the latest cumulative data demonstrated an increase in short-term mortality in STEMI patients during the COVID-19 pandemic as compared to the former year [10]. However, these alarming data were observed only in the Eastern low-middle-income countries. In contrast, longer door-to-balloon time was observed irrespective of the geographical location or national income status [10]. This outcome might draw attention towards low-middle-income countries incapable of adequately confronting the overwhelming effects of the COVID-19 outbreak on STEMI treatment. However, these data should also be interpreted with caution. Foremost, this study compared STEMI patients treated with PCI during and before the outbreak, thus direct comparison between COVID-19 positive and negative subgroups was not performed. Furthermore, there are potential discrepancies associated with different populations, healthcare systems, treatment strategy and endpoint definitions. There was substantial heterogeneity among included centers. All studies involved in this meta-analysis were conducted retrospectively. Thus, straightforward comparison with this outcome might be limited. Our analysis confirmed a longer time from first medical contact to angiography with similar risk of death and periprocedural complications despite the time of intervention. However, intrahospital delay and clinical outcome were evaluated only in the time of the COVID-19 outbreak between positive and negative patients during both on- and off-hours. Thus, direct comparison with data from meta-analyses cannot be provided. Importantly, the sample size might not be adequate to perceive the disparity in mortality and adverse event rates. More common use of potent antiplatelet agents and a higher proportion of final TIMI flow grade 0 or 1 might be linked with enhanced risk of complications in longer observation. There is a paucity of studies evaluating clinical outcomes between COVID-19 positive and negative patients with STEMI regarding the time of intervention. Most contemporary reports before the pandemic suggested an increased risk of death during off-hours [13,14]. Delay in angiography and revascularization, circadian variability in myocardial perfusion or platelet aggregation during off-hours, operator experience and fatigue were presumed risk factors for detrimental outcomes during night hours [13,14]. In this study, a radial expertise and dexterity level were similar in both timeframes. Likewise, there was a similar rate of radial access use across groups. Furthermore, there were no differences in total radiation dose and contrast volume. Thus, an equal mortality rate might be partially explained by the similar competence of invasive cardiologists and the reorganization of healthcare services. However, only short-term outcomes are attainable. Long-term follow-up is essential to assess the effect of COVID-19 on STEMI treatment [30]. All possible efforts should be taken to minimize the total myocardial ischemia time and provide optimal healthcare for STEMI patients during the COVID-19 outbreak [30].

### Limitations

This study has all of the limitations inherent to non-randomized designs. The risk of unmeasured confounding variables cannot be ruled out. However, PSM was calculated to prevail over this limitation. Furthermore, a relatively low sample size might abate the statistical power of the analysis and limit the generalizability of outcomes. Only periprocedural mortality was evaluated, no in-hospital outcome was available. Additionally, post-discharge data were not collected. The long-term observation of patients might be crucial for comprehensive assessment of influence of the COVID-19 outbreak on treatment delay. Some of the high-risk patients might die before transfer to an invasive cardiology center. Furthermore, in 2020, there was a significant decline in the total number of STEMI patients compared to previous years, as some patients might not have sought medical help on time. The higher prevalence of prehospital death cannot be excluded. Thus, the presented analysis cannot cover the entire population with STEMI cured in the time of the study period. Delay in treatment might potentially be influenced by other factors, such as asymptomatic older patients being unable to provide precise information about symptom onset. Furthermore, there is a possibility of preselection bias associated with cross-over across groups in patients admitted during shift change. Irrespective of all these limitations, our study reflected evidence from national perspective from an immense unselected group of patients from both urban and rural areas. Furthermore, healthcare in Poland is supplied by one national institution and the majority of the population is Caucasian. Data from national perspective might be more homogenous and more reliable as compared to meta-analyses. Thus, we presented holistic insights into everyday clinical practice in STEMI patients in the time of COVID-19 outbreak.

## 5. Conclusions

Patients with a positive test for COVID-19 might experience a longer time from first medical contact to angiography. There was no impact of COVID-19 diagnosis on the rate of periprocedural mortality or periprocedural complication irrespective of time of intervention. Public education and systems-level changes might be crucial in minimizing total myocardial ischemia time and improve healthcare of STEMI patients during COVID-19 pandemic.

## Figures and Tables

**Table 1:** Baseline characteristics before propensity score matching.

Variable		On-Hours		*p*-Value	Off-Hours		*p*-Value
		COVID-19 (−) (*n* = 6236)	COVID-19 (+) (*n* = 877)		COVID-19 (−) (*n* = 3817)	COVID-19 (+) (*n* = 418)	
Male gender		4204 (67.6)	666 (76.2)	0.001	2562 (67.3)	305 (73.7)	0.01
Age (years)		65.2 (12.0)	65.4 (11.5)	0.7	64.7 (12.2)	64.9 (13.1)	0.8
Diabetes mellitus		1046 (16.8)	137 (15.6)	0.4	701 (18.4)	89 (21.3)	0.2
Previous stroke		201 (3.2)	22 (2.5)	0.3	116 (3.0)	21 (5.0)	0.04
Previous MI		804 (12.9)	87 (9.9)	0.02	526 (13.8)	53 (12.7)	0.6
Previous CABG		121 (1.9)	11 (1.3)	0.2	60 (1.6)	7 (1.7)	1.0
Previous PCI		789 (12.7)	80 (9.1)	0.003	511 (13.4)	62 (14.8)	0.5
Smoking		2051 (32.9)	217 (24.7)	0.001	1308 (34.3)	128 (30.6)	0.2
Arterial hypertension		3647 (58.5)	410 (46.8)	0.001	2256 (59.1)	238 (56.9)	0.4
Chronic kidney disease		231 (3.7)	27 (3.1)	0.4	129 (3.4)	19 (4.5)	0.3
Chronic obstructive pulmonary disease		159 (2.5)	29 (3.3)	0.2	116 (3.0)	15 (3.6)	0.6
Killip-Kimball Class							
	I	5297 (85)	737 (84)	0.001	3160 (82.5)	352 (84.2)	0.008
	II	601 (9.6)	82 (9.4)	0.001	388 (10.2)	31 (7.4)	0.008
	III	135 (2.2)	26 (3.0)	0.001	132 (3.5)	17 (4.1)	0.008
	IV	203 (3.3)	32 (3.6)	0.001	140 (3.7)	18 (4.3)	0.008
Cardiac arrest at admission		244 (3.9)	180 (20.5)	0.001	151 (4.0)	20 (4.8)	0.5

Data are presented as number (percentage) or mean and standard deviation. CABG—coronary artery bypass grafting; MI—myocardial infarction; PCI—percutaneous coronary intervention.

**Table 2:** Angiographic characteristics after propensity score matching.

Variable	On-Hours		*p*-Value	Off-Hours		*p*-Value
	COVID-19 (−) (*n* = 877)	COVID-19 (+) (*n* = 877)		COVID-19 (−) (*n* = 418)	COVID-19 (+) (*n* = 418)	
Extent of coronary artery disease:						
Single-vessel disease	464 (52.9%)	508 (57.9%)	0.002	222 (53.1%)	221 (52.9%)	0.6
LMCA only	5 (0.6%)	1 (0.1%)	0.002	0 (0.0%)	3 (0.7%)	0.6
Multivessel disease without LMCA	363 (41.4%)	312 (35.6%)	0.002	167 (40.0%)	166 (39.7%)	0.6
Multivessel disease with LMCA	45 (5.1%)	56 (6.4%)	0.002	29 (6.9%)	28 (6.7%)	0.6
TIMI flow before PCI						
0	479 (54.6%)	490 (55.9%)	0.9	254 (60.8%)	233 (55.7%)	0.15
1	129 (14.7%)	121 (13.8%)	0.9	39 (9.3%)	60 (14.4%)	0.15
2	138 (15.7%)	136 (15.5%)	0.9	76 (18.2%)	76 (18.2%)	0.15
3	131 (14.9%)	130 (14.8%)	0.9	49 (11.7%)	49 (11.7%)	0.15
TIMI flow after PCI						
0	16 (1.8%)	25 (2.9%)	0.048	10 (2.4%)	9 (2.2%)	0.6
1	16 (1.8%)	21 (2.4%)	0.048	4 (1.0%)	9 (2.2%)	0.6
2	30 (3.4%)	49 (5.6%)	0.048	25 (6.0%)	25 (6.0%)	0.6
3	815 (92.9%)	782 (89.2%)	0.048	379 (90.7%)	375 (89.7%)	0.6

Data are presented as number (percentage). LMCA—left main coronary artery; PCI—percutaneous coronary intervention; TIMI—thrombolysis in myocardial infarction.

**Table 3:** Percutaneous coronary intervention details after propensity match score.

Variable	On-Hours		*p*-Value	Off-Hours		*p*-Value
	COVID-19 (−) (*n* = 877)	COVID-19 (+) (*n* = 877)		COVID-19 (−) (*n* = 418)	COVID-19 (+) (*n* = 418)	
Site volume ≥400 PCI in current year	728 (83.0)	782 (89.2)	0.001	344 (82.3)	364 (87.1)	0.07
Radial approach during angiography	756 (86.2)	758 (86.4)	0.9	360 (86.1)	349 (83.5)	0.3
Radial approach during PCI	688 (78.4)	721 (82.2)	0.1	334 (79.9)	318 (76.1)	0.2
PCI operator radial experience (PCI during 2014–2020) (number of all procedures)	1091.2 (685.1)	1143.5 (635.3)	0.1	1059.8 (±709.5)	1063.9 (±675.3)	0.9
PCI operator radial experience (2014–2020) (% of all performed PCI)	81% (±17%)	83% (±18%)	0.1	81% (±16%)	80% (±18%)	0.5
Total amount of contrast (mL)	151.2 (72.3)	150.57 (65.6)	0.8	148.7 (66.9)	149.7 (60.7)	0.8
Total radiation dose (mGy)	743.6 (788)	758.98 (600.7)	0.6	697.0 (727.1)	723.8 (715.9)	0.6
Aspiration thrombectomy during PCI	74 (8.4)	63 (7.2)	0.4	46 (11.0)	49 (11.7)	0.8
Rotational atherectomy during PCI	4 (0.5)	2 (0.2)	0.7	0 (0.0)	2 (0.5)	0.5
P2Y12 inhibitors before and during PCI						
Clopidogrel	625 (71.3)	543 (61.9)	0.001	316 (75.6)	299 (71.5)	0.2
Ticagrelor	39 (4.4)	60 (6.8)	0.001	13 (3.1)	9 (2.2)	0.2
Prasugrel	213 (24.3)	274 (31.2)	0.001	89 (21.3)	110 (26.3)	0.2
GPI IIb/IIIa during PCI	708 (80.7)	708 (80.7)	1.0	301 (72.0)	284 (67.9)	0.2
Unfractionated heparin during PCI	838 (95.5)	837 (95.4)		387 (92.6)	390 (93.3)	0.2
Low-molecular-weight heparins during PCI	26 (3.0)	34 (3.9)	0.4	25 (6.0)	21 (5.0)	0.6
Bivalirudin during PCI	13 (1.5)	6 (0.7)	0.2	6 (1.4)	7 (1.7)	1.0

Data are presented as number (percentage) or mean and standard deviation. PCI—percutaneous coronary intervention.

**Table 4:** Periprocedural outcome after propensity score matching.

Variable	On-Hours		*p*-Value	Off Hours		*p*-Value
	COVID-19 (−) (*n* = 877)	COVID-19 (+) (*n* = 877)		COVID-19 (−) (*n* = 418)	COVID-19 (+) (*n* = 418)	
No-reflow	18 (2.1%)	20 (2.3%)	0.9	8 (1.9%)	9 (2.2%)	0.9
Bleeding at the puncture site	15 (1.7%)	7 (0.8%)	0.1	7 (1.7%)	7 (1.7%)	1.0
Allergic reaction	1 (0.1%)	1 (0.1%)	1.0	1 (0.2%)	0 (0.0%)	0.2
Coronary artery perforation	9 (1.0%)	2 (0.2%)	0.1	2 (0.5%)	2 (0.5%)	1.0
Dissection of coronary artery	2 (0.2%)	2 (0.2%)	1.0	2 (0.5%)	1 (0.2%)	0.9
Cardiac arrest	19 (2.2%)	17 (1.9%)	0.8	9 (2.2%)	12 (2.9%)	0.5
Periprocedural stroke	1 (0.1%)	0 (0.0%)	0.7	0	0	-
Any periprocedural complication	42 (4.8%)	33 (3.8%)	0.3	19 (4.5%)	22 (5.3%)	0.7
Periprocedural death	17 (1.9%)	11 (1.3%)	0.3	4 (1.0%)	7 (1.7%)	0.5

**Table 5:** Treatment delays after propensity score matching.

Variable	On-Hours		*p*-Value	Off Hours		*p*-Value
	COVID-19 (−) (*n* = 877)	COVID-19 (+) (*n* = 877)		COVID-19 (−) (*n* = 418)	COVID-19 (+) (*n* = 418)	
Time from pain to first medical contact, minutes	230.1 (294.5)	235.6 (296.1)	0.7	214.2 (261.4)	212.8 (264.1)	0.9
Time from pain to angiography, minutes	340.2 (317.2)	360.8 (323.3)	0.2	323.12 (296.6)	356.5 (332.9)	0.1
Time from first medical contact to angiography, minutes	117.1 (135.8)	133.8 (137.1)	0.01	112.2 (138.7)	148.1 (201.6)	0.003
Time from first medical contact to angiography, <90 min	490 (55.9)	353 (40.3)	0.001	252 (60.3)	203 (48.6)	0.001
Time from first medical contact to angiography, <120 min	625 (71.3)	439 (50.1)	0.001	312 (74.6)	276 (66.0)	0.008
